# Soil bacterial community diversity, composition, and species specificity across different geographical landscapes in the Mu Us Sandy Land

**DOI:** 10.3389/fmicb.2025.1714794

**Published:** 2025-12-08

**Authors:** Xue Shang, Zhaoquan He, Wenbo Chen, Xiaoze Jin

**Affiliations:** 1 Office of Information Technology, Yan'an University, Yan'An, China; 2 School of Life Sciences, Yan'an University, Yan'An, China; 3 Shaanxi Key Laboratory of Research and Utilization of Resource Plants on the Loess Plateau, College of Life Sciences, Yan'an University, Yan'An, China; 4 Key Laboratory of Applied Ecology of Universities in Shaanxi Province on the Loess Plateau, Yan'an University, Yan'An, China; 5 Northwest Institute of Eco-Environment and Resources, Chinese Academy of Sciences, Lanzhou, China; 6 National Key Laboratory of Uranium Resource Exploration-Mining and Nuclear Remote Sensing, East China University of Technology, Nanchang, China

**Keywords:** soil microbiome, 16S rRNA gene, high-throughput sequencing, ecological restoration, arid region ecosystem

## Abstract

The Mu Us Sandy Land represents a typical region for ecological restoration in China, characterized by the development of diverse landscapes including desert, meadow patches, arbor forests, and mixed arbor-shrub forests. This study aimed to investigate the diversity, composition, and differential taxa of soil bacterial communities across these distinct geographical landscapes, thereby elucidating the driving mechanisms of vegetation restoration on the sandy land soil microbiome. Soil samples were collected from four typical landscapes in the Mu Us Sandy Land: desert (B), meadow patch (D), arbor forest (T), and mixed arbor-shrub forest (C). High-throughput sequencing of the 16S rRNA gene was performed using the Illumina NextSeq 2000 platform. Our results revealed distinct patterns of bacterial community composition: Actinobacteria dominated the desert (37.42%), while Proteobacteria were more abundant in meadow patches and mixed arbor-shrub forests, and *Bacillota* were significantly enriched in arbor forests (20.32%). Beta diversity analysis combined with the ANOSIM test (*R* = 0.7168, *P* = 0.001) revealed significant divergence in bacterial community structure among the different landscapes. LEfSe analysis further identified specific biomarkers for each landscape, such as *Rubrobacter* and *Streptomyces* in the desert, and taxa associated with *Acidobacteria* and Proteobacteria in the mixed arbor-shrub forests. The research demonstrates that the different geographical landscapes in the Mu Us Sandy Land shape distinct soil bacterial communities. The mixed arbor-shrub forest exhibited a more complex community structure compared to the pure arbor forest, indicating its potential as a more sustainable and resilient ecological restoration model. These findings provide a baseline understanding of microbial community shifts associated with vegetation restoration, which may inform future studies integrating soil physicochemical drivers.

## 1 Introduction

The Mu Us Sandy Land, as one of China's four major sandy lands, represents not only a typical ecologically fragile region but also stands out as one of the most remarkable areas globally in terms of achievements in desertification control and ecological restoration (Chen et al., 2022). Long-term efforts in sand fixation have resulted in the formation of a diverse mosaic of geographical landscapes in this region, including deserts, meadow patches, pure stands of arbor species (such as poplar and Mongolian Scots pine), and mixed arbor-shrub forests. Soil microorganisms, particularly bacteria, act as the “core engine” of ecosystem functioning, driving critical belowground processes such as organic matter decomposition, nutrient cycling, and maintaining ecosystem stability (Seitz et al., 2021; Wang and Li, 2023). Therefore, systematically elucidating the response characteristics of soil bacterial communities across different geographical landscapes at various restoration stages is of great theoretical and practical significance for deepening our understanding of the microbiological mechanisms underlying sandy land ecosystem restoration, assessing the long-term benefits of ecological restoration projects, and guiding future vegetation restoration practices (Sanaullah et al., 2011; Li et al., 2021).

In recent years, significant progress has been made in research on the effects of vegetation restoration on soil microbial communities (Han et al., 2023; He et al., 2024). Numerous studies have demonstrated that during the ecological succession from desert to forest, as vegetation cover increases and soil physicochemical properties improve (e.g., increases in organic carbon and nitrogen content), the α-diversity of soil bacteria (e.g., Chao1 and Shannon indices) generally shows a significant increasing trend (Zhang et al., 2023; Zuo et al., 2023). In terms of community composition, drought-tolerant and oligotrophic phyla such as Actinobacteria and Cyanobacteria tend to dominate in desert ecosystems, while the relative abundances of phyla associated with organic matter decomposition and complex nutrient cycling, such as Proteobacteria, Acidobacteria, and Planctomycetes, gradually increase as environmental conditions improve (Campbell and Kirchman, 2012; Song et al., 2025). These previous studies have established a fundamental theoretical framework suggesting that changes in microbial communities are a response to the comprehensive improvement of the plant-soil system driven by vegetation restoration.

However, a thorough comparative analysis of existing research reveals notable limitations and theoretical controversies in this field. Firstly, most studies focus on simple binary comparisons between “desert and woodland” or linear succession sequences, paying insufficient attention to meadow patches with unique ecological functions that coexist within the ecosystem, as well as the mixed arbor-shrub forest model widely promoted in current ecological restoration practices (Hu et al., 2021). This limited perspective may lead to an incomplete understanding of the mechanisms maintaining microbial diversity in sandy land ecosystems. Secondly, previous research has predominantly emphasized changes in community diversity and composition at the phylum and class levels, with relatively scarce in-depth analysis at finer taxonomic resolutions such as the genus level and below. This gap constrains our ability to identify key functional taxa and their ecological roles (Zhao et al., 2019; Zhang et al., 2024). Thirdly, there is ongoing debate regarding the long-term stability of microbial communities under specific landscape types, such as pure arbor forests: while some argue that arbor forests maximize the promotion of microbial diversity and function, others suggest that the “biological filtering effect” induced by single-species stands or specific alterations in soil physicochemical properties (e.g., acidification, imbalanced carbon-to-nitrogen ratios) may suppress certain microbial taxa, resulting in diversity levels even lower than those in more structurally complex mixed arbor-shrub forests (Wang et al., 2022; Zhu et al., 2025). These controversies underscore the urgency of conducting refined comparative studies across different geographical landscapes.

The aforementioned limitations collectively point to a critical research gap: it remains unclear whether there are significant differentiations in the characteristics of soil bacterial communities (from diversity to compositional structure) among the distinct geographical landscapes—formed through both natural recovery and human intervention and exhibiting significant functional differences (desert, meadow patches, arbor forest, mixed arbor-shrub forest)—in the Mu Us Sandy Land, and what mechanisms drive such differentiations (Landesman et al., 2014; Jiang et al., 2023; Dong et al., 2024). Addressing this question is crucial for evaluating the microbial effects of different ecological restoration models.

Based on this, the present study aims to systematically investigate the characteristics of soil bacterial communities in four typical geographical landscapes (desert, meadow patches, arbor forest, mixed arbor-shrub forest) within the Mu Us Sandy Land. Utilizing high-throughput sequencing technology, we focus on analyzing: (1) differences in community Beta diversity; (2) patterns of change in community composition from the phylum to genus level; and (3) identifying characteristic taxa indicative of different landscapes through differential species analysis. This research will provide a scientific basis from a microbial ecology perspective for assessing the sustainability of different ecological restoration models in the Mu Us Sandy Land, reveal the potential advantages of the mixed arbor-shrub forest model in maintaining soil microbial diversity and ecosystem functioning, and thereby offer theoretical guidance for future desertification control and ecological management practices in China.

## 2 Materials and methods

### 2.1 Study area

This study was conducted in the Mu Us Sandy Land (37°27.5′N−39°22.5′N, 107°20′E−111°30′E), located in the transitional zone between the Ordos Plateau and the Loess Plateau. The area has an average elevation of 1,200–1,600 m above sea level, characterized predominantly by fixed and semi-fixed dunes, interspersed with mobile dunes, lake basins, and denuded ridges. The region experiences a temperate semi-arid continental monsoon climate, with a mean annual precipitation ranging from 250 to 440 mm, decreasing from southeast to northwest. Precipitation exhibits high interannual variability and is concentrated primarily between July and September (Garg et al., 2025). The mean annual temperature ranges from 6.0 °C to 8.5 °C, while the mean annual potential evaporation is high, between 2,000 and 2,500 mm. The predominant soil type is Aeolian sandy soil, found mainly on various dunes. These soils are characterized by infertility, loose structure, very low organic matter content, weak pedogenesis, poor water and nutrient retention capacity, and high susceptibility to wind and water erosion. Key soil properties include a pH of approximately 8.0, total nitrogen content of 0.2–0.8 g kg^−1^, available phosphorus of 2–5 mg kg^−1^, and a bulk density of 1.54 g cm^−3^. The native vegetation consists mainly of psammophytic and xerophytic herbs and shrubs, such as Artemisia ordosica, Caragana korshinskii, Salix psammophila, and Leymus chinensis, forming simple community structures (Gao et al., 2022).

### 2.2 Landscape ecological characteristics of the typical geographical landscapes

The desert landscape in the Mu Us Sandy Land is characterized by a matrix of mobile, semi-fixed, and fixed dunes with impoverished Aeolian sandy soil exhibiting extremely poor water retention. Annual precipitation ranges from 250 to 450 mm, coupled with intense evaporation, making water availability the sole direct limiting factor. Patch edges are persistently sculpted by wind, resulting in highly irregular shapes, low fragmentation but high dynamism. The dominant vegetation comprises super-xerophytic shrubs, semi-shrubs, and pioneer herbs: shrubs like Caragana korshinskii, Hedysarum scoparium, Salix psammophila, Salix cheilophila, and Artemisia desertorum can root in mobile dunes, while short-lived herbs such as Agriophyllum squarrosum and Psammochloa villosa complete their life cycles during brief wet periods, collectively forming the primary vegetation of the desert system (Zhang et al., 2025).

Meadow patches are distributed in interdune depressions or low-lying areas with a shallow water table depth of 0.5–1.5 m. The soils are meadow soils or mildly salinized meadow soils, with seasonal waterlogging contributing to relatively high productivity. These patches are typically small, nearly circular in shape, and discretely embedded among dunes, with edges subject to dual disturbances from grazing and salinization. Dominant species are hygro-mesophytic and salt-tolerant herbs: Achnatherum splendens forms dense tussocks, while species like Potentilla anserina, Ligularia virgaurea, and Anemone rivularis grow in zones along micro-topographic salinity gradients, serving as core contributors to soil nitrogen accumulation in these lowlands.

Arbor forests are found only in areas with the most favorable water conditions, such as low-lying areas or the edges of fixed dunes, relying on groundwater or artificial irrigation. They often exhibit a “stunted tree” phenomenon due to uncontrolled planting density. Plantation patches are typically regular and rectangular, whereas remnant natural forest patches show high fragmentation and low fractal dimension. Dominant species differ by origin: plantations primarily consist of conifers like Pinus tabuliformis and Pinus sylvestris var. mongolica, aimed at rapid sand fixation and timber production, while remnant natural forests are characterized by broadleaf species such as Betula albosinensis and Betula platyphylla (Huang et al., 2025).

Mixed arbor-shrub forests represent the climax community on fixed dunes, featuring a shrub layer covering 60%−80% and sparse trees covering less than 20%, effectively stabilizing parabolic dunes. The system achieves efficient water redistribution through vertical niche separation, with deep-rooted shrubs utilizing groundwater and shallow-rooted trees utilizing capillary water. These patches are extensively contiguous and exhibit the lowest fragmentation. Artemisia ordosica and Caragana korshinskii constitute the absolutely dominant shrubs, associated with Salix psammophila and Hippophae rhamnoides. The tree layer primarily consists of naturally regenerated Juniperus rigida and Ephedra sinica, forming a self-sustaining, continuous sand-fixing belt capable of intercepting wind and sand (Wang et al., 2023).

### 2.3 Research methods

#### 2.3.1 Experimental design and soil sampling

We selected four landscapes: desert (B), meadow patch (D), arbor forest (T), and mixed arbor-shrub forest (C). Six replicate plots per landscape were established. In June 2025, within each plot, five sampling points were selected along an “S”-shaped transect. After removing surface litter, soil samples were collected from the 0–20 cm depth using a sterile soil sampler. Soil samples from the same plot were thoroughly homogenized into one composite sample, immediately placed into sterile zip-lock bags, and rapidly transported to the laboratory on dry ice. Each composite sample was divided into two aliquots and stored at −80 °C in an ultra-low-temperature freezer for subsequent extraction of total soil microbial DNA and high-throughput sequencing (Tian et al., 2022).

#### 2.3.2 Soil DNA extraction and high-throughput sequencing

Total genomic DNA was extracted from 0.5 g of the soil sample using the E.Z.N.A.^®^ soil DNA Kit (Omega Bio-tek, Norcross, GA, United States). The integrity of the extracted DNA was checked by 1% agarose gel electrophoresis, and its concentration and purity were determined using a NanoDrop2000 spectrophotometer (Thermo Scientific, United States; Ganzert et al., 2014).

Qualified DNA served as the template for PCR amplification of the V3–V4 hypervariable region of the bacterial 16S rRNA gene using barcoded specific primers 338F (5′-ACTCCTACGGGAGGCAGCAG3′) and 806R (5′-GGACTACHVGGGTWTCTAAT3′). The PCR reaction mixture (20 μl) contained: 4 μl of 5 × TransStart FastPfu Buffer, 2 μl of 2.5 mM dNTPs, 0.8 μl each of forward and reverse primers (5 μM), 0.4 μl of TransStart FastPfu DNA Polymerase, 10 ng of template DNA, and ddH_2_O up to 20 μl. The amplification program was: 95 °C for 3 min; 27 cycles of 95 °C for 30 s, 55 °C for 30 s, and 72 °C for 45 s; followed by a final extension at 72 °C for 10 min (Crits-Christoph et al., 2020). PCR products were verified by 2% agarose gel electrophoresis and purified using a PCR Clean-Up Kit (YuHua, China). Paired-end sequencing (PE250) was performed on an Illumina Nextseq2000 platform (Majorbio Bio-Pharm Technology Co., Ltd., Shanghai, China). The raw sequencing data have been deposited in the NCBI SRA database.

#### 2.3.3 Data processing and bioinformatics analysis

**Figure 1:**
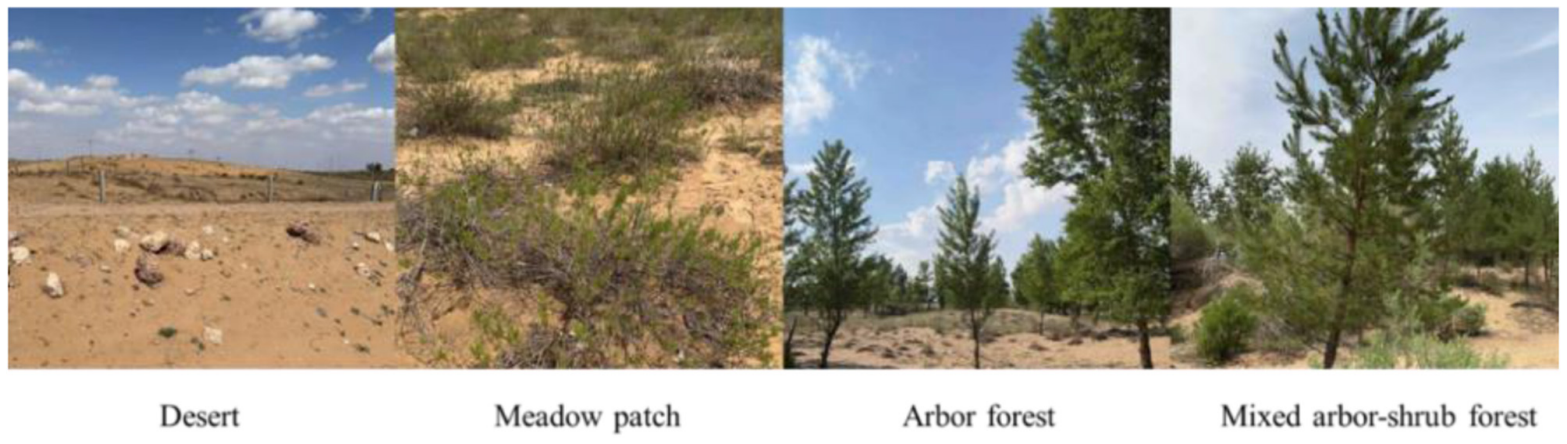
The distinct geographical landscapes.

Raw sequencing data were processed on the Majorbio Cloud Platform (https://cloud.majorbio.com). Quality control and filtering of raw reads were performed using fastp (v0.19.6), and paired-end reads were merged using FLASH (v1.2.11). Raw sequences were processed using QIIME2 (v2020.2). DADA2 was used with a quality threshold of *Q*-score ≥20 for denoising and ASV generation. The DADA2 plugin within the QIIME2 (v2020.2) pipeline was then used for denoising the quality-filtered sequences with the following parameters: –p-trunc-len-f 240, –p-trunc-len-r 200, –p-max-ee 2.0, to generate amplicon sequence variants (ASVs) at 100% similarity (Yin et al., 2024). Quality control and filtering of raw reads were performed using fastp (v0.19.6) with default parameters to remove low-quality sequences (*Q*-score < 20, length < 150 bp). Sequences identified as originating from mitochondria or chloroplasts were removed. To ensure fairness in subsequent diversity analyses, the sequence count for all samples was rarefied to 20,000 reads per sample. Rarefaction curves are provided in Figure 1, confirming that sequencing depth was sufficient to capture the majority of bacterial diversity. After rarefaction, the Good's coverage index exceeded 99% for all samples. Taxonomic annotation of ASVs was performed using the Naive Bayes classifier against the SILVA database (v138).

#### 2.3.4 Statistical analysis

Beta diversity was assessed using Principal Coordinates Analysis (PCoA) based on both Bray-Curtis dissimilarity and weighted UniFrac distance, Principal Component Analysis (PCA), and Non-metric Multidimensional Scaling (NMDS). Permutational multivariate analysis of variance (PERMANOVA; Adonis) was used to test the significance of differences in community structure among groups. Linear discriminant analysis Effect Size (LEfSe; LDA score >2, with the alpha value for the factorial Kruskal-Wallis test set at 0.05) was employed to identify significantly differentially abundant taxa (biomarkers) from phylum to genus level across the different landscapes.

## 3 Results

### 3.1 ASV analysis

A systematic analysis of soil bacterial community diversity was conducted across four typical geographical landscapes in the Mu Us Sandy Land: desert, meadow patch, arbor forest, and mixed arbor-shrub forest. Based on 16S rRNA gene data obtained via Illumina Nextseq 2000 platform sequencing and processed through the QIIME2 pipeline, a total of 50,235 high-quality sequences per sample were obtained on average. Amplicon Sequence Variant (ASV) analysis revealed differences in bacterial richness among the landscape types. The arbor forest sample T5 exhibited the highest number of ASVs (2,736), while the desert and some mixed arbor-shrub forest (C2: 1,812) and arbor forest (T2: 1,819) samples showed lower ASV numbers, indicating higher bacterial species richness in sites with a higher degree of vegetation restoration (Table 1).

**Table 1:** Statistics of ASV effective sequences.

**Sample**	**ASV_num**	**Seq_num**
C3	2,330	50,235
C2	1,812	50,235
C1	2,020	50,235
C6	2,052	50,235
C5	2,379	50,235
C4	2,364	50,235
T6	2,314	50,235
T4	2,461	50,235
T5	2,736	50,235
T2	1,819	50,235

Sample_info, sample information; ASV_number, number of ASVs per sample; Seq_num, number of effective sequences per sample.

### 3.2 Community diversity analysis

**Figure 2:**
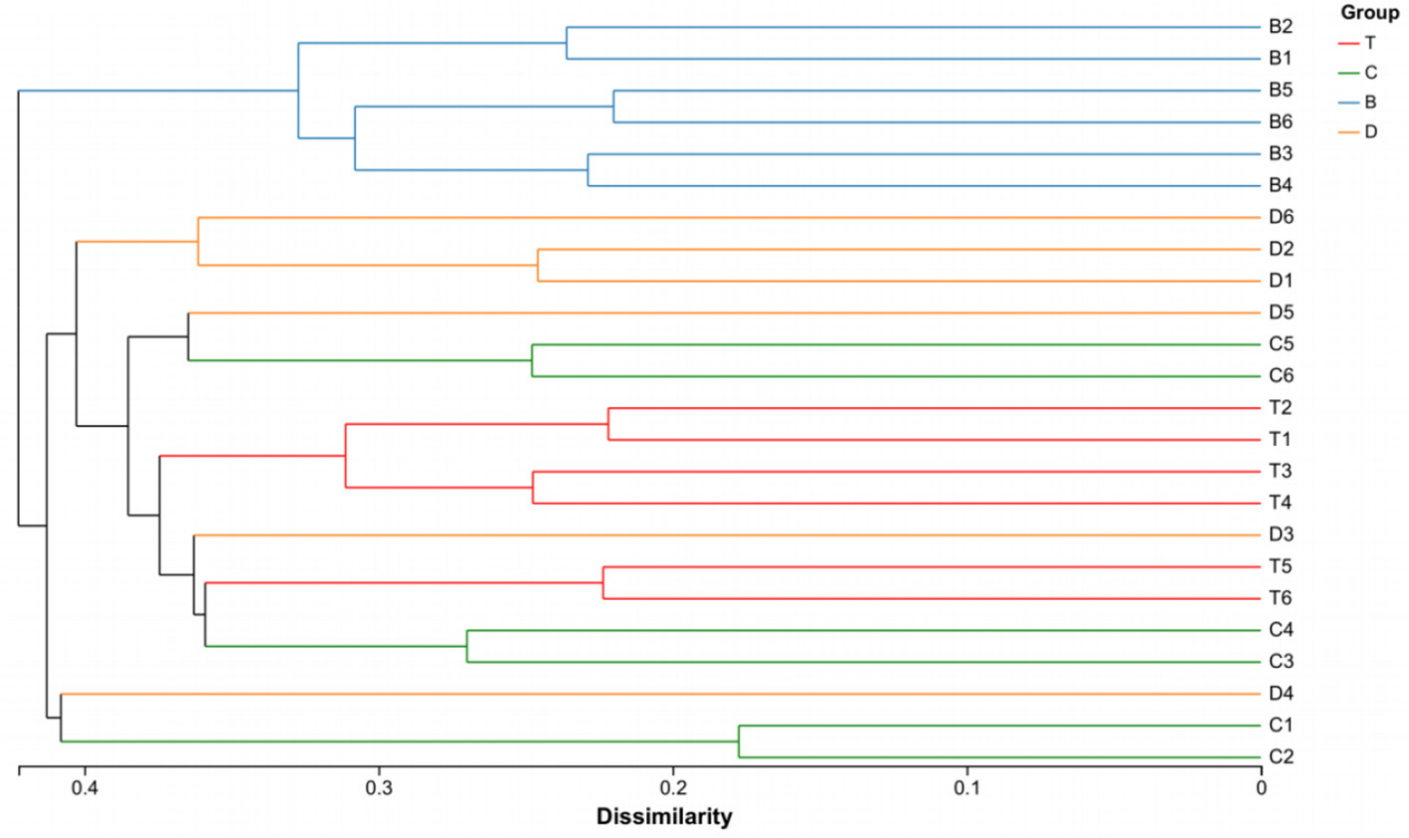
Hierarchical clustering tree of samples. The branch lengths represent the distances between samples; different groups can be shown in different colors. When the stacked bar chart is displayed, the composition of the dominant taxa in each sample is shown on the right side of the clustering tree. B, desert; D, meadow patch; T, arboreal forest; C, tree–shrub mixed forest.

**Figure 3:**
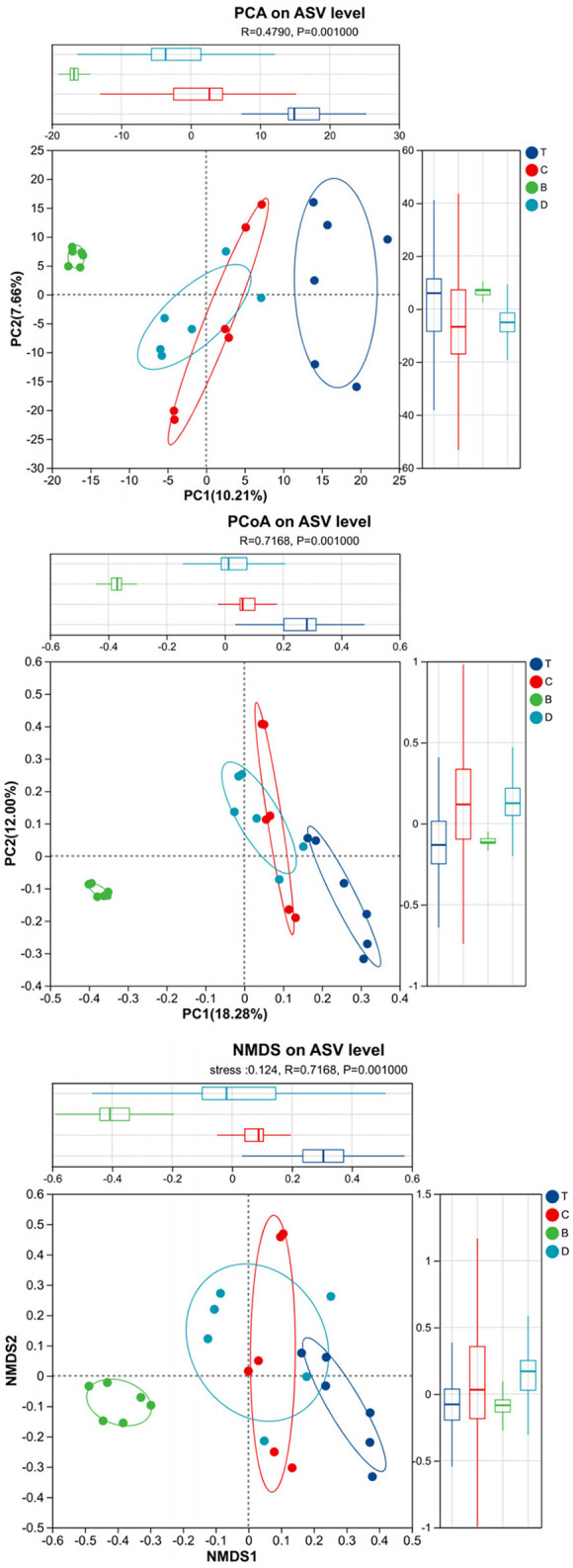
PCA, PCoA, and NMDS scatter plots. The horizontal and vertical axes represent the two selected principal coordinates; the percentages indicate the contribution of each coordinate to the overall dissimilarity among samples. The axis scales show relative distances and have no absolute meaning. Points of different colors or shapes denote samples from different groups; the closer two points are, the more similar their species compositions. B, desert; D, meadow patch; T, arboreal forest; C, tree–shrub mixed forest.

Beta diversity analysis, employing multiple methods, revealed significant divergence in community structure. Hierarchical clustering analysis (UPGMA) showed that samples clustered according to landscape type. Desert samples were clearly separated from the other three landscape types. A certain degree of separation was also observed among meadow patch, arbor forest, and mixed arbor-shrub forest samples, although some overlap occurred between mixed arbor-shrub forest and arbor forest samples, suggesting a degree of similarity in their community structures (Figure 2). Principal Component Analysis (PCA) showed that the first two principal components (PC1 and PC2) explained 10.21 and 7.86% of the variance, respectively. The low cumulative variance explained by PCA (PC1 + PC2 < 20%) may reflect the high heterogeneity of soil microbial communities in arid ecosystems, or the absence of measured environmental variables that explain additional variation. Although the explained variance was relatively low, a separation trend among samples from different landscapes was still evident, with desert samples distinctly separated from the other three types. Principal Coordinates Analysis (PCoA) based on Bray-Curtis distance revealed that PC1 and PC2 explained 18.28 and 12.00% of the variance, respectively. PERMANOVA further confirmed significant differences between groups (Bray-Curtis: *R* = 0.7168, *P* = 0.001; Weighted UniFrac: *R* = 0.6985, *P* = 0.001). Non-metric Multidimensional Scaling (NMDS) analysis (Stress = 0.124) yielded results consistent with PCoA, and the ANOSIM test indicated that inter-group differences were significantly greater than intra-group differences (*R* = 0.7168, *P* = 0.001), demonstrating that desert, meadow patches, arbor forests, and mixed arbor-shrub forests each possessed distinct soil bacterial community structures (Figure 3).

### 3.3 Community composition analysis

**Figure 4:**
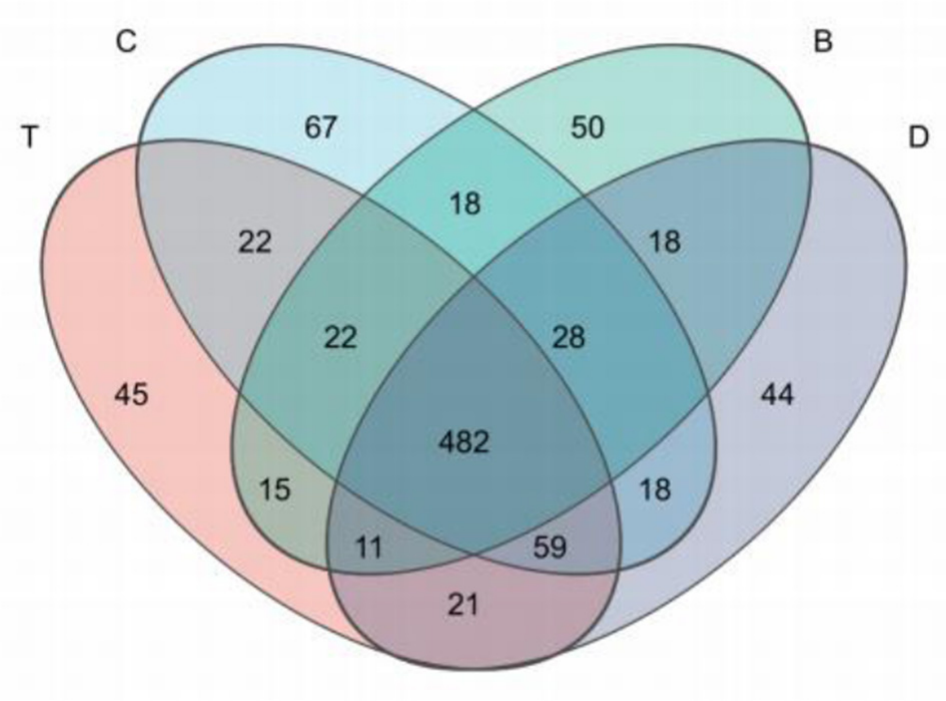
Venn diagram. Each color represents a different group (or sample). Overlapping areas indicate species shared by multiple groups (or samples), while non-overlapping areas indicate species unique to a single group (or sample). The numbers show the corresponding species counts. B, desert; D, meadow patch; T, arboreal forest; C, tree–shrub mixed forest.

Venn diagram analysis showed that the number of ASVs unique to each of the four landscapes (desert, meadow patch, arbor forest, mixed arbor-shrub forest) was relatively high, while the number of shared ASVs was low. This indicated a high degree of specificity and differentiation among the soil bacterial communities under different geographical landscapes (Figure 4), further supporting the conclusion of significant community structure divergence observed in the beta diversity analysis.

**Figure 5:**
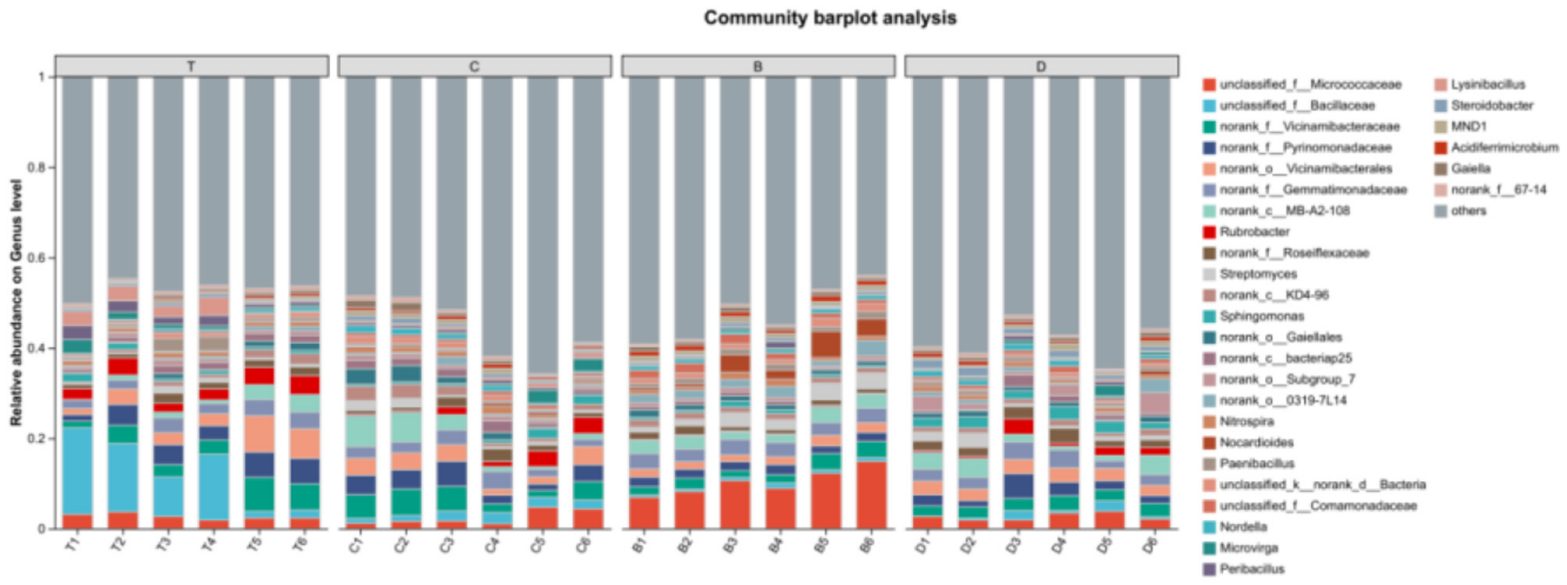
Community bar plot analysis at the phylum level across the four landscape types. The *x*-axis (or *y*-axis) lists the sample names; the *y*-axis (or *x*-axis) shows the relative proportion of each taxon within the sample. Bars of different colors represent different taxa, and bar length reflects the proportion of each taxon. Group labels are displayed above the bars for each grouping. B, desert; D, meadow patch; T, arboreal forest; C, tree–shrub mixed forest.

Bar plots illustrating community composition at the phylum level showed the relative abundance trends of major bacterial phyla across the landscapes. Actinobacteria were the absolutely dominant phylum in the desert (average 37.42%), reflecting its adaptation to arid and oligotrophic conditions. The abundance of Proteobacteria significantly increased in meadow patches and mixed arbor-shrub forests, reaching 25.64 and 21.59%, respectively, suggesting that bacterial groups associated with nutrient cycling gradually became dominant with vegetation restoration (Figure 5). The proportion of *Bacillota* (a phylum that encompasses many Gram-positive bacteria and has superseded the name “Firmicutes” in recent taxonomic frameworks) in arbor forests was significantly higher than in other landscapes (20.32%), potentially related to specific soil conditions in this landscape type.

**Figure 6:**
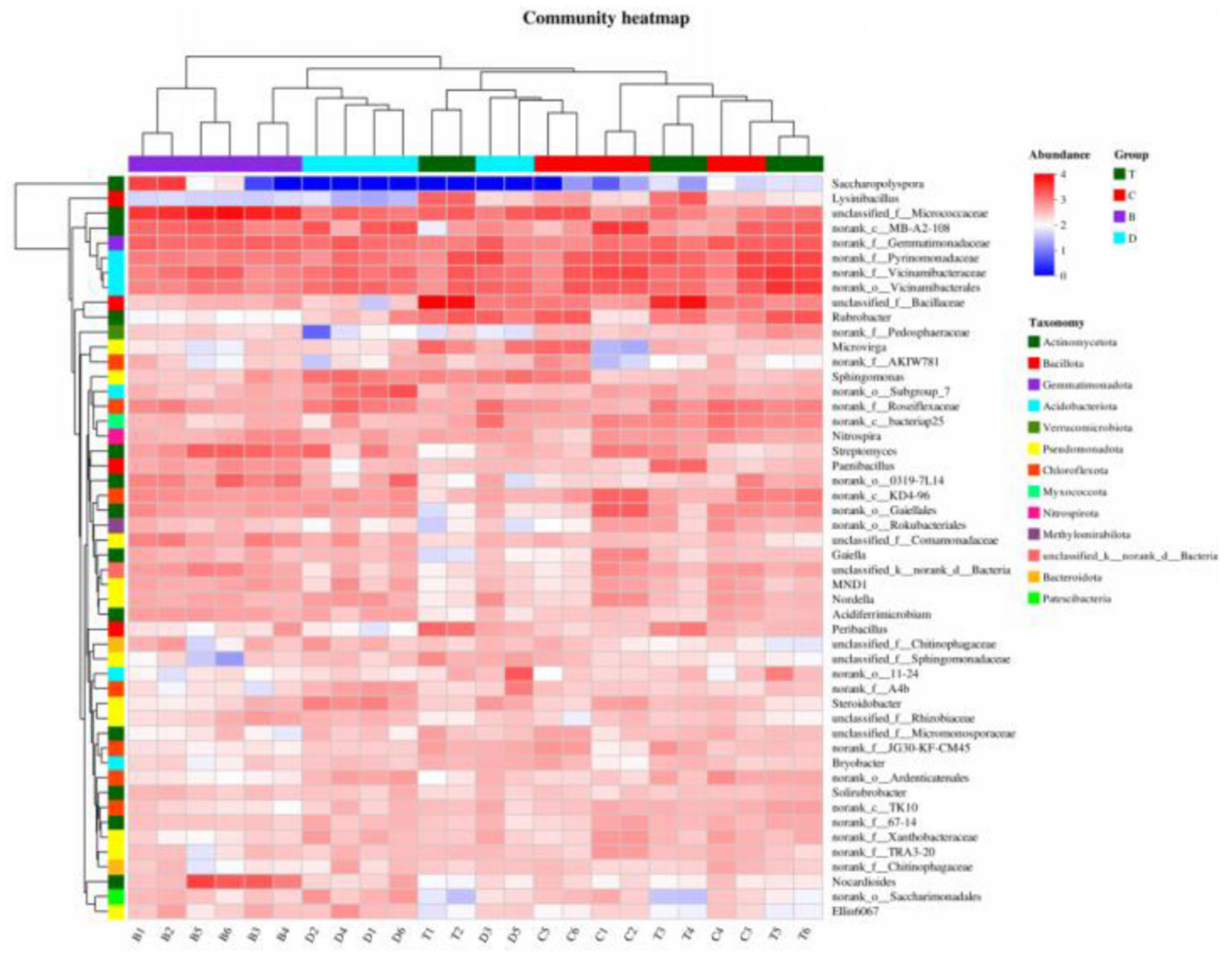
Heatmap showing the distribution of dominant bacterial genera across samples. The x-axis shows sample names, and the y-axis shows species names. A color gradient indicates the relative proportion of each species within each sample; the scale bar on the right maps colors to values. B, desert; D, meadow patch; T, arboreal forest; C, tree–shrub mixed forest.

Heatmap analysis further revealed the distribution patterns of bacterial taxa at the genus level across different samples. The color gradients indicated significant enrichment of drought-tolerant genera like *Rubrobacter* and *Streptomyces* in desert samples, whereas meadow and mixed arbor-shrub forest samples showed higher abundances of genera associated with organic matter decomposition, such as *Sphingomonas* (Figure 6). Specific taxa within the phylum *Bacillota* were uniquely enriched in arbor forest samples, consistent with the bar plot results.

**Figure 7:**
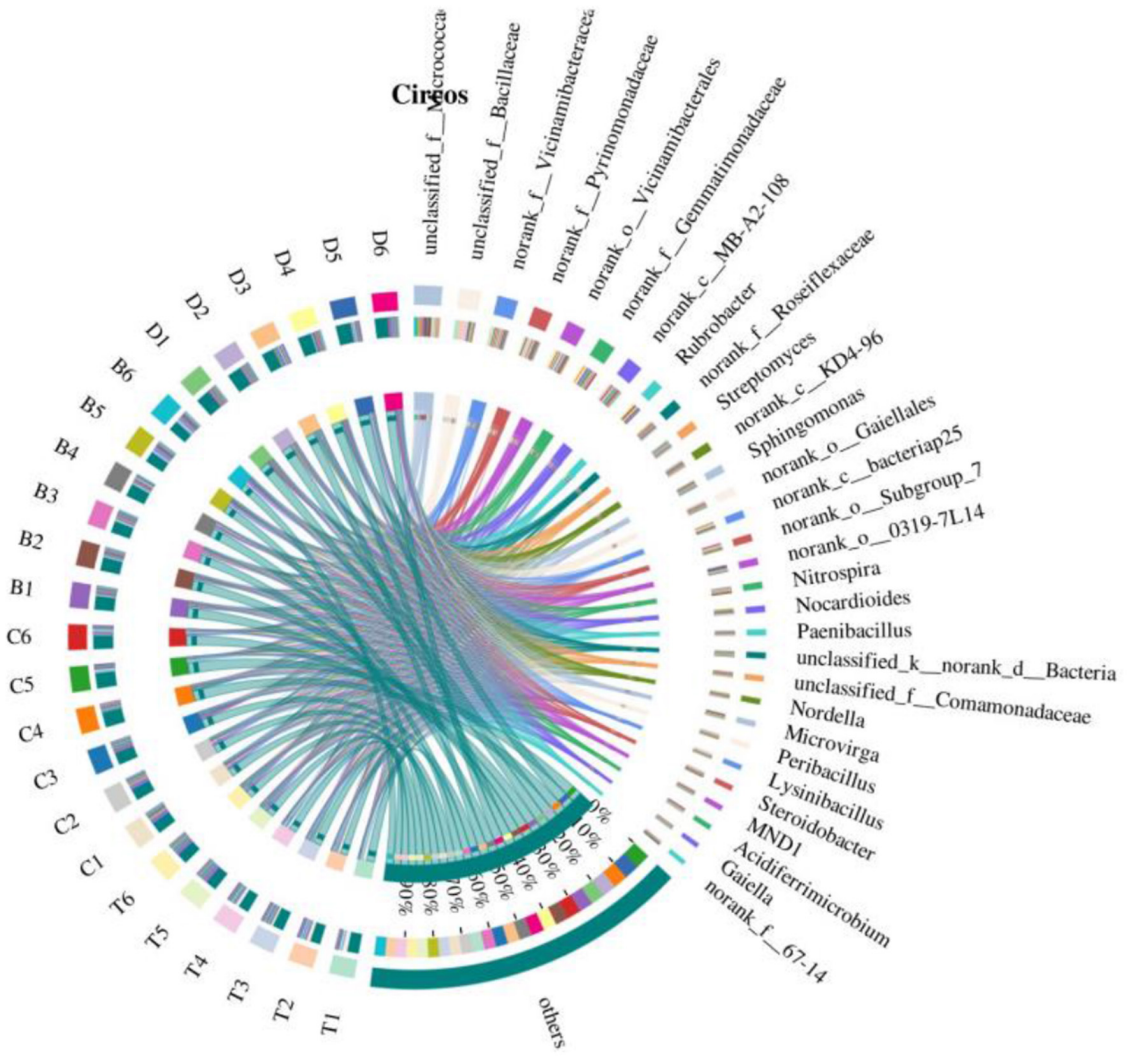
Circos diagram. The left semicircle displays the species composition within each sample. The outer ribbon color indicates the group of origin, while the inner ribbon color denotes the species; ribbon length reflects the species' relative abundance in that sample. The right semicircle shows how each taxon is distributed across samples at the chosen taxonomic level: the outer ribbon represents the species, the inner ribbon color indicates the group, and ribbon length represents the proportion of the species contributed by each sample. B, desert; D, meadow patch; T, arboreal forest; C, tree–shrub mixed forest.

Circos diagrams visually reinforced the correspondence between samples and bacterial phyla. The ribbon connections showed strong associations between Actinobacteria and desert samples, while Proteobacteria and *Acidobacteria* were more linked to meadow and mixed arbor-shrub forest samples. Arbor forests showed a stronger connection with *Bacillota*. This diagram intuitively reflected the structural differences in bacterial community composition across the different landscape types (Figure 7).

In summary, the different geographical landscapes in the Mu Us Sandy Land significantly influenced the composition and structure of soil bacterial communities. From desert to vegetation-restored areas, the bacterial community transitioned from drought-tolerant, oligotrophic taxa toward copiotrophic taxa associated with organic matter decomposition and nutrient cycling. The mixed arbor-shrub forest exhibited a more complex bacterial community structure than the pure arbor forest, suggesting it might possess higher ecological stability and functional diversity.

### 3.4 Differential species analysis

**Figure 8:**
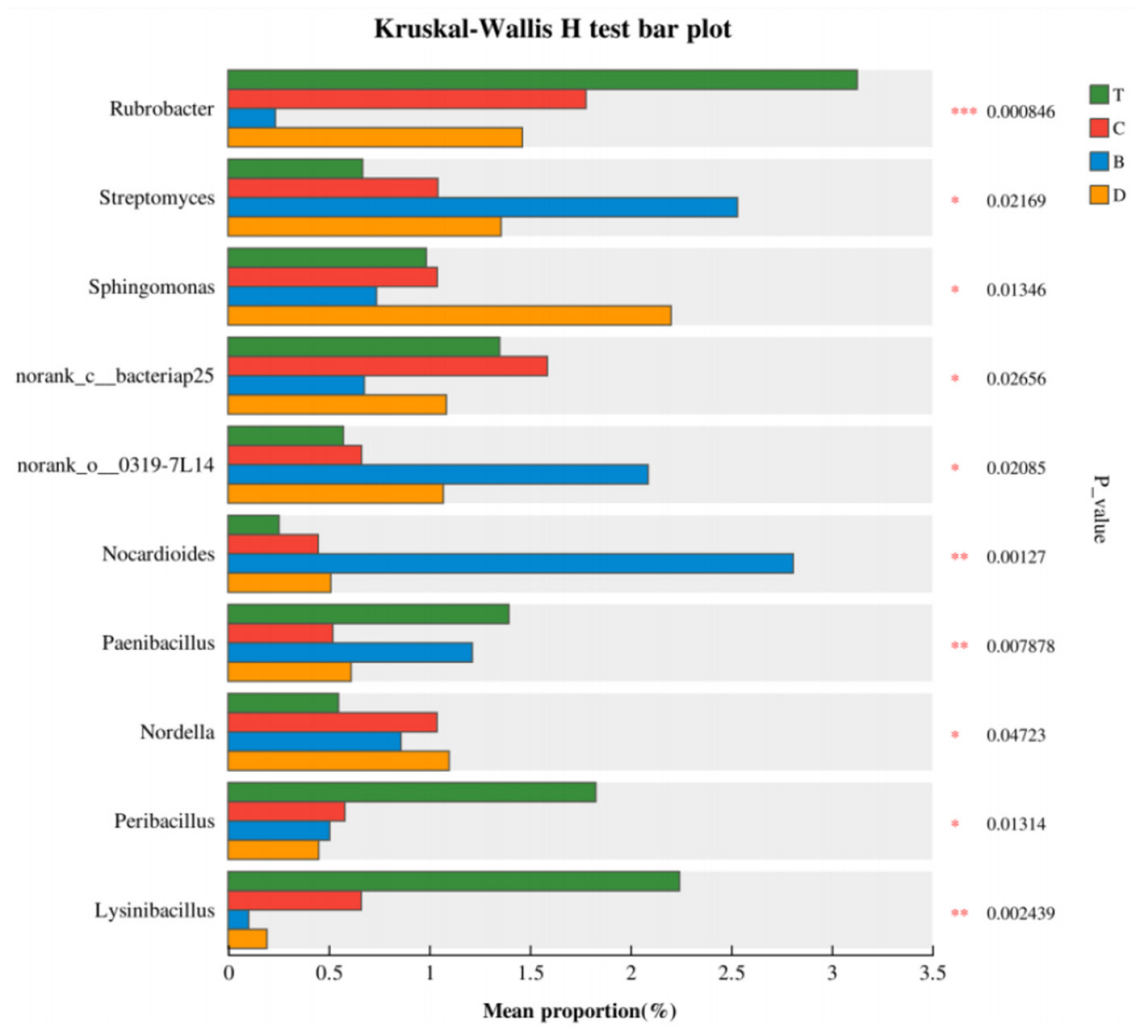
Kruskal–Wallis test plot showing significantly different genera across the four landscape types. The *y*-axis lists taxon names at the indicated taxonomic level; the *x*-axis shows the percent relative abundance of each taxon. Different colors represent different groups. *P*-values are given on the far right: 0.01 < *P* ≤ 0.05, 0.001 < *P* ≤ 0.01, *P* ≤ 0.001. B, desert; D, meadow patch; T, arboreal forest; C, tree–shrub mixed forest.

Differential species analysis further identified key indicator taxa (biomarkers) across the landscapes. A multi-group comparison analysis (Kruskal-Wallis test, *P* < 0.05) identified 10 significantly different species at the genus level (Figure 8). Among them, *Rubrobacter* and *Streptomyces* (both belonging to *Actinobacteria*) were significantly enriched in the desert. *Sphingomonas* (*Proteobacteria*) was distributed across multiple groups but with significant abundance differences. Genera like *Norcardioides, Paenibacillus*, and *Lysinibacillus* showed specific distributions in particular landscapes.

**Figure 9:**
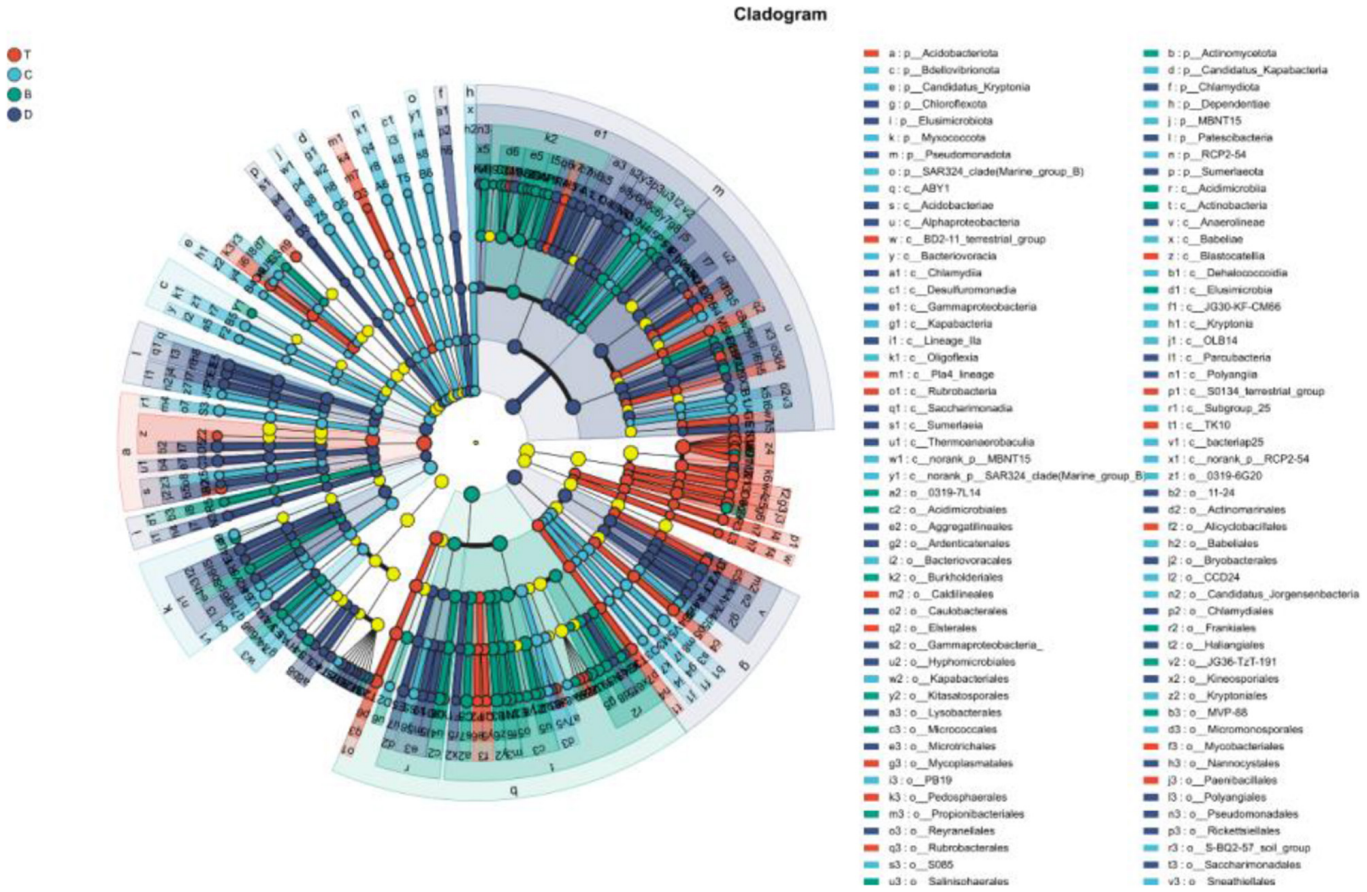
LEfSe multi-level cladogram. Colored nodes indicate microbial clades that are significantly enriched in the corresponding group and contribute to the observed inter-group differences; pale-yellow nodes represent clades without significant enrichment in any group or without a significant effect on inter-group differentiation. B, desert; D, meadow patch; T, arboreal forest; C, tree–shrub mixed forest.

**Figure 10:**
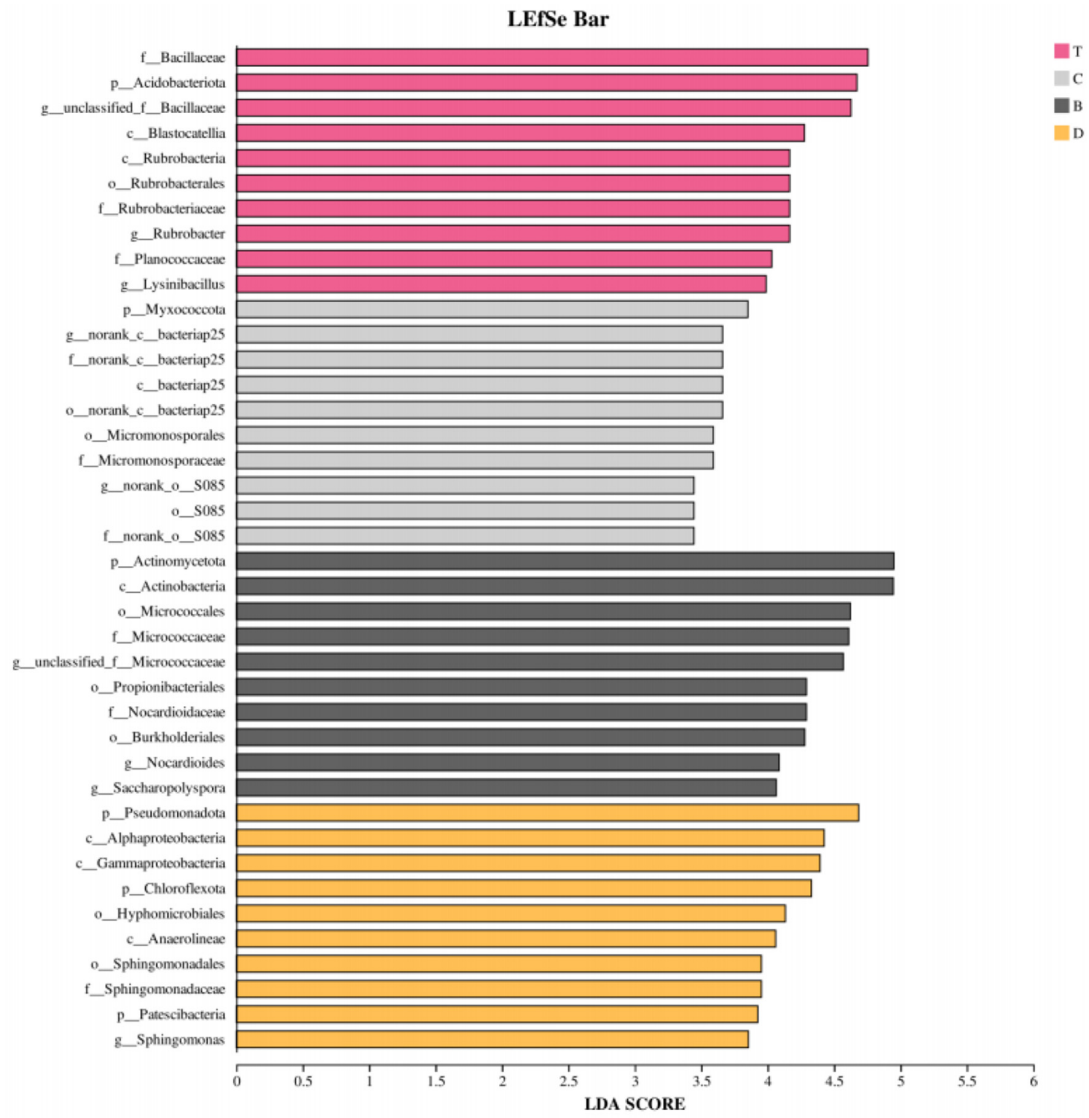
LEfSe analysis identifying differentially abundant taxa (LDA score >4). The larger the LDA score, the greater the contribution of the taxon's abundance to the observed group difference. B, desert; D, meadow patch; T, arboreal forest; C, tree–shrub mixed forest.

Linear Discriminant Analysis Effect Size (LEfSe) analysis further identified unique biomarkers across multiple taxonomic levels for each group (LDA score >4; Figures 9, 10). Signature taxa for the desert included *Rubrobacter, Rubrobacteraceae*, and *Micrococcaceae*. Meadow patches were associated with certain taxa within the phylum *Chloroflexi*. Biomarkers for arbor forests were related to the phylum *Bacillota*. The signature taxa for mixed arbor-shrub forests included multiple families and genera within the phyla *Acidobacteria* and Proteobacteria. These differential taxa indicated a shift in the soil bacterial community from drought-tolerant, oligotrophic groups in the desert toward more diverse groups closely associated with organic matter decomposition and nutrient cycling in the well-vegetated arbor forests and mixed arbor-shrub forests.

This multi-faceted bioinformatics analysis demonstrated that the soil bacterial communities in the different geographical landscapes of the Mu Us Sandy Land exhibited significant differences in terms of diversity, structural composition, and functional groups. The vegetation restoration model, particularly the mixed arbor-shrub forest, displayed a more complex bacterial community structure compared to the pure arbor forest, which may have important implications for the stability of its ecosystem functioning. These findings provide crucial data support for evaluating the microbial effects of different ecological restoration models.

## 4 Discussion

This study systematically revealed significant differences in the diversity, composition, and functional groups of soil bacterial communities across four typical geographical landscapes in the Mu Us Sandy Land using high-throughput sequencing technology. It not only confirms the profound impact of vegetation restoration on the soil micro-ecosystem from a microbial ecology perspective but also provides an important microbiological basis for evaluating the effectiveness of different ecological restoration models (Zhao et al., 2020).

### 4.1 Succession of soil bacterial community structure and function across different geographical landscapes in the Mu Us Sandy Land

This study observed a directional succession in bacterial community structure associated with vegetation restoration. The harsh environmental conditions (infertility, aridity) of the desert ecosystem shaped a community structure overwhelmingly dominated by the phylum Actinobacteria (Xu et al., 2018). This aligns strongly with the “environmental filtering” theory in microbial ecology, where stringent abiotic stresses (e.g., low moisture, low organic matter) select for oligotrophic microorganisms possessing traits like dormant forms (e.g., actinobacterial spores), high stress tolerance, and the ability to degrade recalcitrant organic matter (Ren et al., 2022; Wiryawan et al., 2022). Genera such as *Rubrobacter* and *Streptomyces*, identified as signature biomarkers for the desert, are typical representatives of this strategy.

In stark contrast, in the meadow patches, arbor forests, and mixed arbor-shrub forests that have undergone vegetation restoration, increased input of plant litter and improved soil microenvironments led to elevated soil organic carbon and nutrient availability (Li M. et al., 2022; Li Y. T. et al., 2022; Yoon et al., 2024). The notable enrichment of *Bacillota* (a phylum name that has largely replaced “Firmicutes” in updated taxonomic databases and includes many Gram-positive bacteria) in pure arbor forests may be linked to their physiological traits, such as spore-forming capabilities, which confer resilience to environmental fluctuations or specific root exudate profiles (Zhao et al., 2020). This drove a shift in the bacterial community toward copiotrophic taxa. The significant increase in the relative abundance of phyla like *Proteobacteria* and *Acidobacteria*, which are closely involved in carbon and nitrogen cycling, signifies an enhancement of ecosystem functioning—a transition from mere stress survival toward more active nutrient cycling and energy flow (Xu et al., 2021). This pattern of change is consistent with findings from global studies on vegetation restoration-driven succession of soil microbial communities.

### 4.2 Characteristics of ecological restoration models in the Mu Us Sandy Land

A key finding of this study is the differential impact of various vegetation restoration models on the microbial community. The notably high relative abundance of *Bacillota* in pure arbor forests composed of a single tree species might be related to soil acidification or imbalanced C/N ratios induced by the monoculture. This “biological filtering effect” could lead to a simplification of functional groups, potentially affecting the long-term stability of the ecosystem (Shi et al., 2022; Zhou et al., 2025). The phylum *Bacillota* comprises many Gram-positive bacteria, which may become enriched in certain disturbed environments or under specific selective pressures (e.g., root exudates from a single tree species, potential soil acidification). This phenomenon suggests that monoculture afforestation may exert a specific “selection effect” on the soil microbial community, causing its functional profile to become less diverse, which could potentially impact the long-term stability and resistance of the ecosystem (Yoon et al., 2024).

In comparison, the mixed arbor-shrub forest exhibited higher bacterial species richness (ASV numbers) and a more complex community structure, with its signature biomarkers encompassing various taxa within *Acidobacteria* and *Proteobacteria*. This higher diversity may be linked to the “diversity-stability” hypothesis. The mixed arbor-shrub forest, incorporating both shrubs and trees, likely creates more diverse ecological niches through its greater variety of litter inputs, root architectures, and root exudates (Wei et al., 2018). This, in turn, supports a more functionally diverse bacterial community, potentially rendering the ecosystem more resilient to environmental fluctuations. This indicates that for ecological management in the Mu Us Sandy Land, adopting near-natural restoration models combining trees and shrubs may be more conducive to the health and stability of the soil micro-ecosystem compared to single-species afforestation (Su et al., 2020).

### 4.3 Study limitations and future perspectives

In conclusion, this study demonstrates, at the microbial scale, that the ecological restoration process in the Mu Us Sandy Land is accompanied by a succession of soil bacterial communities from oligotrophic to copiotrophic strategies. It highlights that compared to single-species afforestation, mixed arbor-shrub forests foster a more diverse and complex soil bacterial community, suggesting greater potential for ecosystem functionality. The mixed arbor-shrub forest exhibited higher bacterial richness and more complex community structure, which may support greater functional redundancy and potential resilience (Xu et al., 2021).

However, this study has certain limitations. Firstly, the lack of concurrent soil physicochemical data (e.g., pH, SOC, TN) prevents a direct mechanistic interpretation of the observed microbial shifts. Future studies should integrate such measurements with microbial profiling. Secondly, our analysis was confined to bacterial communities based on 16S rRNA gene sequencing. A more holistic ecosystem understanding would require the characterization of other key microbial groups, such as fungi and archaea, and their interactions. Furthermore, the potential confounding effects of environmental variables like microclimate (moisture, temperature) across the landscapes, while acknowledged, were not quantified. Thirdly, while the “space-for-time substitution” approach is valuable, long-term temporal monitoring is essential to validate successional dynamics. Future work should also employ metagenomic and metatranscriptomic approaches to move beyond taxonomic composition and directly link the observed community shifts to changes in functional gene abundance and expression, thereby providing direct evidence of ecosystem functional changes (Li M. et al., 2022; Li Y. T. et al., 2022). Phylogenetic analyses beyond taxonomy, such as inferring community assembly processes, could also offer deeper ecological insights.

## 5 Conclusions

Based on the analysis of soil bacterial community characteristics across four typical geographical landscapes in the Mu Us Sandy Land, this study draws the following main conclusions:

- Geographical landscape type is a key determinant of soil bacterial community structure and diversity in the Mu Us Sandy Land. The desert, meadow patches, arbor forests, and mixed arbor-shrub forests each harbored significantly distinct bacterial communities.
- Bacterial communities underwent a directional succession along with vegetation restoration. The community structure shifted from being dominated by drought-tolerant, oligotrophic phyla such as *Actinobacteria* in the desert toward a predominance of copiotrophic phyla like *Proteobacteria* in vegetated restoration areas.
- Different vegetation restoration models exerted differential effects on the microbial community. Compared to single-species arbor forests, the mixed arbor-shrub forest fostered a bacterial community with higher complexity and potential functional diversity, indicating it is a restoration model with greater micro-ecological benefits.
- Each landscape type possessed specific microbial biomarkers (e.g., *Rubrobacter* in the desert; *Acidobacteria* in the mixed arbor-shrub forest). These biomarkers hold potential as microbial indicators for assessing the status of ecological restoration and soil health in the Mu Us Sandy Land.

In summary, this study demonstrates at the microbial scale that adopting a mixed arbor-shrub restoration model in the ecological management of the Mu Us Sandy Land is likely more conducive to the health and stability of the soil micro-ecosystem compared to single-species afforestation. These findings provide important theoretical significance for guiding future desertification control practices and ecological benefit assessments.

## Data Availability

The data presented in the study are deposited in the NCBI repository, accession number PRJNA1370616.
